# High-Quality Draft Genome Sequence and Characterization of ProBC Plus *Weizmannia* (*Bacillus*) *coagulans* LMG S-31876

**DOI:** 10.1128/mra.01018-22

**Published:** 2022-12-06

**Authors:** Ranjith Kumar Kallur, Sreenadh Madapati, Ankita Mathur

**Affiliations:** a Abode Biotec India Private Limited, Hyderabad, Telangana, India; Wellesley College

## Abstract

The whole-genome sequence of a *Weizmannia* (*Bacillus*) *coagulans* (ProBC Plus) strain isolated from fermented rice is reported here. The complete genome analysis of the strain will be helpful in the future to combat multitudinous problems and will be helpful in providing insights regarding potential probiotic properties.

## ANNOUNCEMENT

The strain was isolated from fermented rice samples and cultured in de Man-Rogosa-Sharpe (MRS) medium for 24 to 48 h at 37°C. The 16S rRNA gene region was amplified using the forward primer 27F 5′ (AGA GTT TGA TCM TGG CTC AG) 3′ and the reverse primer 1492R 5′ (TAC GGY TAC CTT GTT ACG ACT T) 3′. The gene was sequenced by the Sanger dideoxy sequencing method at Macrogen (Seoul, South Korea). The strain exhibited 100% similarity to Weizmannia coagulans LMG S-31876 (GenBank accession number MZ687045) (1).

Genomic DNA from a culture pellet was fragmented with the phenol-chloroform method (2) and sonicated in separate, generic, polypropylene tubes on a Qsonica Q800R2 sonicator. One hundred nanograms of fragmented DNA was used to prepare a paired-end sequencing library with the NEBNext Ultra II DNA library preparation kit (Illumina), and libraries were sequenced on an Illumina MiSeq instrument using the MiSeq reagent kit v2 (500 cycles).

A total of 1,75,28,596 paired-end reads of 150-bp read length on average, with genome coverage of 782.52×, were sequenced. Of these, 1,49,85,902 high-quality paired-ended reads were filtered with the next-generation sequencing (NGS) quality control (QC) Toolkit, with a Phred quality score cutoff value of 20 for high-quality filtering (3). The high-quality paired-end reads were assembled into 183 contigs with the *de novo* genome assembler SPAdes v3.15.5 (4). The draft genome consists of 183 contigs containing 3,461,262 bp, with a GC content of 46% and an *N*_50_ value of 64,268 bp; the largest assembled scaffold is 214,117 bp (3). The genome sequence was annotated with the NCBI Prokaryotic Genome Annotation Pipeline (PGAP) v6.2 (5). The genes were predicted and translated with the Prodigal program (6), following pathway identification with the KEGG Automatic Annotation Server (KAAS) (7). A total of 3,388 genes, 3,289 coding sequences (CDSs), 17 rRNAs, and 77 tRNAs were predicted. A total of 140 genes are involved in the synthesis of proteins and enzymes needed for dormancy and sporulation stages, and the strain is projected to encode approximately 324 proteins involved in carbohydrate metabolism and 264 proteins involved in amino acid metabolism. The strain also has genes for biotin, riboflavin, cobalamin, thiamine, vitamin B_6_, and folate production.

The genome was screened to determine putative virulence factors (with the Virulence Factor Database [VFDB] [8]), plasmids (with PlasmidFinder v2.0 [9]), and antibiotic resistance genes (with the Antibiotic Resistance Genes Database [ARDB] [10]). There was an absence of any plasmids or antibiotic resistance genes, and genes encoding putative virulence factors such as the hemolytic enterotoxin complex HBL (*hblA*, *hblB*, *hblC*, and *hblD*), nonhemolytic enterotoxin (NHE) (*nheA*, *nheB*, and *nheC*), and cytotoxin K (hemolysin IV) were not found. BLASTx comparisons were performed between the Weizmannia coagulans LMG S-31876 draft genome and biogenic-amine-producing protein sequences, and genes such as those for histidine decarboxylase (*hdc*), tyrosine decarboxylase (*tdc*), ornithine decarboxylase, agmatine dihydrolase (deiminase), and putrescine were absent (11). CRISPRFinder was used to screen for clustered regularly interspaced short palindromic repeat (CRISPR) sequences, and Weizmannia coagulans LMG S-31876 contained one confirmed CRISPR sequence (12). Analysis of the genome sequence of ProBC Plus Weizmannia coagulans LMG S-31876 indicates that it meets probiotic safety criteria on the genome level.

### Data availability.

The whole-genome shotgun sequence of Weizmannia coagulans LMG S-31876 has been deposited in DDBJ/EMBL/GenBank under the accession number JANKOH000000000. The version described in this paper is the first version, JANKOH010000000. The raw sequence reads have been submitted to the NCBI SRA with accession number SRR21429472.
